# Effects of semaglutide with and without concomitant SGLT2 inhibitor use in participants with type 2 diabetes and chronic kidney disease in the FLOW trial

**DOI:** 10.1038/s41591-024-03133-0

**Published:** 2024-06-24

**Authors:** Johannes F. E. Mann, Peter Rossing, George Bakris, Nicolas Belmar, Heidrun Bosch-Traberg, Robert Busch, David M. Charytan, Samy Hadjadj, Pieter Gillard, José Luis Górriz, Thomas Idorn, Linong Ji, Kenneth W. Mahaffey, Vlado Perkovic, Søren Rasmussen, Roland E. Schmieder, Richard E. Pratley, Katherine R. Tuttle

**Affiliations:** 1 KfH Kidney Centre, München, Germany; 2 University Hospital Erlangen, Friedrich Alexander University, Erlangen, Germany; 3 Steno Diabetes Center Copenhagen, Herlev, Denmark; 4 Department of Clinical Medicine, University of Copenhagen, Copenhagen, Denmark; 5 Department of Medicine, AHA Comprehensive Hypertension Center, University of Chicago Medicine, Chicago, IL USA; 6 Novo Nordisk A/S, Søborg, Denmark; 7 Albany Medical Center Division of Community Endocrinology, Albany, NY USA; 8 Nephrology Division, Department of Medicine, New York University Grossman School of Medicine, and NYU Langone Health, New York, NY USA; 9 L’Institut du Thorax, CHU Nantes, CNRS, INSERM, Nantes Université, Nantes, France; 10 Department of Endocrinology, University Hospitals Leuven - KU Leuven, Leuven, Belgium; 11 Department of Nephrology, Hospital Clínico Universitario de Valencia (INCLIVA), Valencia, Spain; 12 Department of Medicine, Universitat de València, Valencia, Spain; 13 Department of Endocrinology and Metabolism, Peking University People’s Hospital, Beijing, China; 14 Stanford Center for Clinical Research, Department of Medicine, Stanford School of Medicine, Palo Alto, CA USA; 15 Faculty of Medicine and Health, University of New South Wales, Sydney, New South Wales Australia; 16 Department of Nephrology and Hypertension, University Hospital Erlangen, Friedrich Alexander University, Erlangen, Germany; 17 AdventHealth Translational Research Institute, Orlando, FL USA; 18 Division of Nephrology, University of Washington, Seattle, WA USA; 19 Providence Medical Research Center, Providence Inland Northwest Health, Spokane, WA USA

**Keywords:** Cardiovascular diseases, Chronic kidney disease, Randomized controlled trials

## Abstract

People with type 2 diabetes and chronic kidney disease have a high risk for kidney failure and cardiovascular (CV) complications. Glucagon-like peptide-1 receptor agonists and sodium-glucose cotransporter-2 inhibitors (SGLT2i) independently reduce CV and kidney events. The effect of combining both is unclear. FLOW trial participants with type 2 diabetes and chronic kidney disease were stratified by baseline SGLT2i use (*N* = 550) or no use (*N* = 2,983) and randomized to semaglutide/placebo. The primary outcome was a composite of kidney failure, ≥50% estimated glomerular filtration rate reduction, kidney death or CV death. The risk of the primary outcome was 24% lower in all participants treated with semaglutide versus placebo (95% confidence interval: 34%, 12%). The primary outcome occurred in 41/277 (semaglutide) versus 38/273 (placebo) participants on SGLT2i at baseline (hazard ratio 1.07; 95% confidence interval: 0.69, 1.67; *P* = 0.755) and in 290/1,490 versus 372/1,493 participants not taking SGLT2i at baseline (hazard ratio 0.73; 0.63, 0.85; *P* < 0.001; *P* interaction 0.109). Three confirmatory secondary outcomes were predefined. Treatment differences favoring semaglutide for total estimated glomerular filtration rate slope (ml min^−1^/1.73 m^2^/year) were 0.75 (−0.01, 1.5) in the SGLT2i subgroup and 1.25 (0.91, 1.58) in the non-SGLT2i subgroup, *P* interaction 0.237. Semaglutide benefits on major CV events and all-cause death were similar regardless of SGLT2i use (*P* interaction 0.741 and 0.901, respectively). The benefits of semaglutide in reducing kidney outcomes were consistent in participants with/without baseline SGLT2i use; power was limited to detect smaller but clinically relevant effects. ClinicalTrials.gov identifier: NCT03819153.

## Abstract (web summary)

In a prespecified analysis of the FLOW trial, the use of an SGLT2 inhibitor did not impact the overall benefits of semaglutide on kidney and cardiovascular outcomes in participants with type 2 diabetes and chronic kidney disease.

## Main

People with type 2 diabetes (T2D) and those with chronic kidney disease (CKD) exhibit a high risk for kidney failure and CV complications. Therefore, it is of global interest to examine potential benefits and adverse effects of glucose-lowering drugs in people with T2D and CKD to prevent death, kidney failure and CV complications. Early trials with glucagon-like peptide-1 receptor agonists (GLP-1 RAs) and with sodium-glucose cotransporter-2 inhibitors (SGLT2i) in persons with T2D and high CV risk were powered for CV outcomes as primary and kidney outcomes as secondary^1,2^. Some of those trials demonstrated benefits on CV outcomes and suggested kidney benefit of both drug classes. Later, three independent trials with SGLT2i in persons with and without T2D and CKD defined kidney outcomes as primary and demonstrated substantial kidney benefits of SGLT2i^3^. Recently, the FLOW (Evaluate Renal Function with Semaglutide Once Weekly) trial was the first dedicated kidney outcomes trial in participants with T2D and CKD that examined a GLP-1 RA, namely once-weekly subcutaneous semaglutide (1.0 mg). A clear benefit of treatment with semaglutide on kidney outcomes was shown as well as on CV outcomes and survival compared with placebo^4^.

SGLT2i are particularly effective in preventing kidney, heart failure and CV outcomes. Notably, GLP-1 RAs, including semaglutide, also provide substantial benefits for CV and CKD outcomes, death^1,2^ and heart failure^5^. Since the molecular mechanisms of action of both drug classes on the CV system and the kidney may be complementary and largely independent of glucose lowering^6^, it has been speculated that the combination might further improve clinical outcomes. Therefore, GLP-1 RAs may become another pillar of therapy for diabetic kidney disease along with inhibitors of the renin–angiotensin system (RASi), mineralocorticoid-receptor antagonists and SGLT2i^7^.

There are no clinical trials that have directly examined the combination of GLP-1 RAs plus SGLT2i on major kidney and CV outcomes in participants with T2D and CKD. Therefore, in a prespecified analysis, we investigated the potential benefits and safety of semaglutide, stratified by baseline use of SGLT2i, in the FLOW trial and also analyzed the effect of SGLT2i use initiated after randomization.

## Results

### Participant characteristics

**Extended Data Fig. 1:**
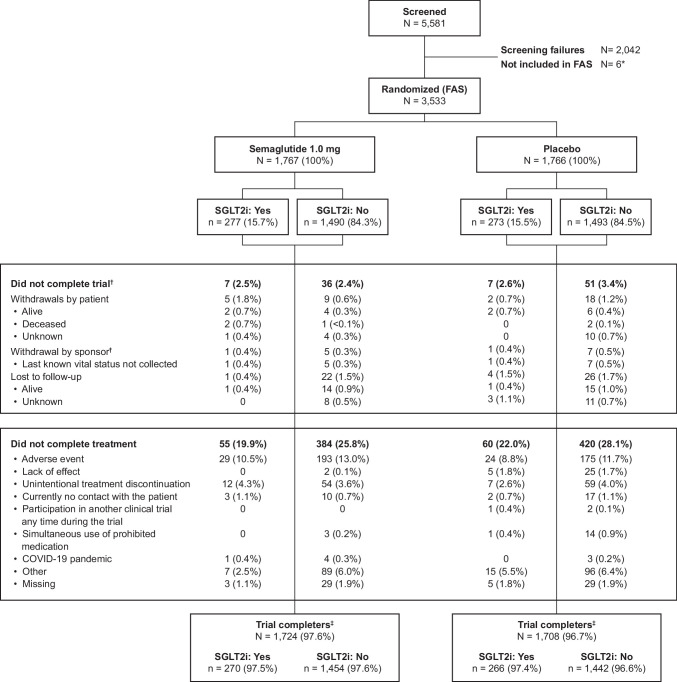
Participant disposition and flow. *Five participants were randomized more than once, and one participant was excluded due to good clinical practice issues at the site. ^†^Fourteen additional withdrawals (two participants receiving semaglutide and twelve receiving placebo) were by sponsor decision, following closure of two sanctioned sites in Russia. ^‡^Participants who attended the follow-up visit or died during the trial. FAS, full analysis set; SGLT2i, sodium-glucose co-transporter-2 inhibitors.

**Extended Data Fig. 2:**
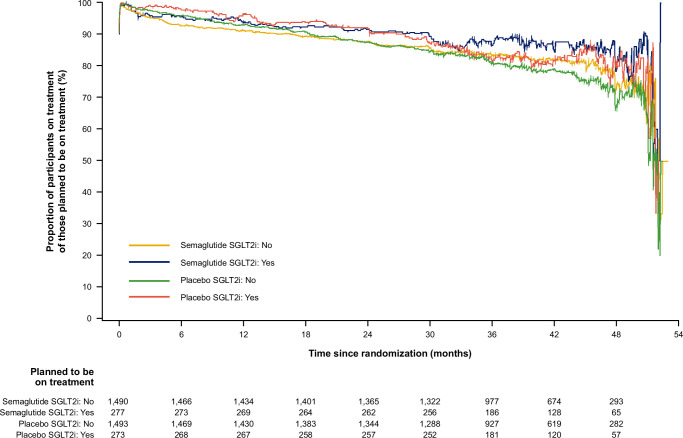
Compliance on randomized treatment over time by baseline SGLT2i use (participants planned to be on treatment). The lines represent the proportion of participants compliant on treatment until the planned end-of-treatment visit. Compliance on-treatment period: all days from the date of first dose to the end of the in-trial period where a dose has been administered. The period could include non-consecutive time intervals, and the dose did not have to be the target dose. SGLT2i, sodium-glucose co-transporter-2 inhibitors.

**Extended Data Fig. 3:**
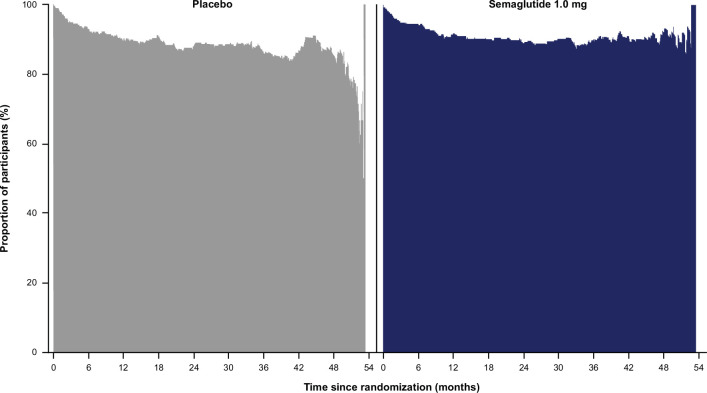
Proportion of participants on SGLT2i at baseline who continued on SGLT2i during the trial. Proportions are based upon the number of participants in the trial at a given time point. SGLT2i, sodium-glucose co-transporter-2 inhibitors.

**Extended Data Fig. 4:**
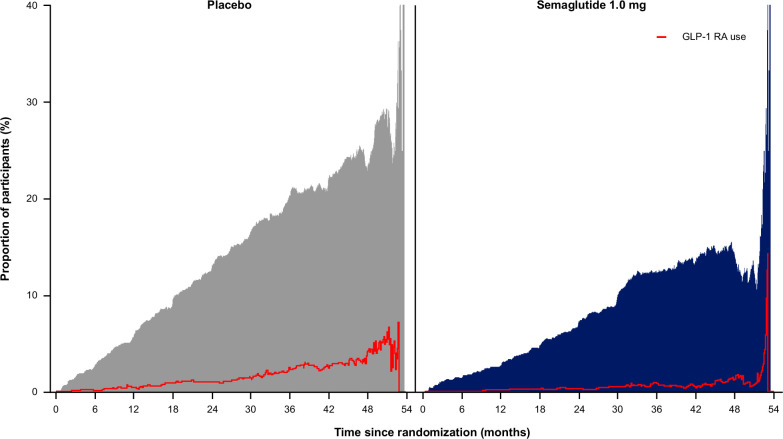
Proportion of participants not on SGLT2i at baseline who started on SGLT2i during the trial (grey and blue areas). Proportions are based upon the number of participants in the trial at a given time point. The red line indicates open-label use of GLP-1 RA. GLP-1 RA, glucagon-like peptide-1 receptor agonist; SGLT2i, sodium-glucose co-transporter-2 inhibitors.

We screened 5,581 participants between June 2019 and May 2021 and randomized 3,533 participants (mean estimated glomerular filtration rate (eGFR) of 47.0 ml min^−1^/1.73 m^2^, median urine albumin‐to-creatinine ratio (UACR) 568 mg g^−1^, mean glycated hemoglobin (HbA_1c_) 7.8%); 550 (15.6%) participants reported SGLT2i use at baseline—277 in the semaglutide and 273 in the placebo group, respectively. Vital status was confirmed for 540 (98.2%) participants reporting SGLT2i use at baseline and for 2,942 (98.6%) not reporting SGLT2i use at baseline; participant flow through the trial is shown in Extended Data Fig. 1. There were no major imbalances between groups for baseline participant characteristics (Supplementary Table 1). Those reporting SGLT2i use tended to be younger, less frequently female, with higher eGFR and lower systolic blood pressure. Glucose-lowering medication use in those reporting SGLT2i use versus no use were comparable (insulin in 57% versus 62%, DPP4 inhibitors 32% versus 25% and sulfonylureas in 25% versus 25%), except for metformin (69% versus 49% with and without SGLT2i use, respectively). RASi were taken by 97% of participants reporting and by 95% of those not reporting SGLT2i use. Adherence to the trial treatment regimen was reported for 89% of the planned time during the study period (Extended Data Fig. 2); permanent discontinuation of randomized treatment was reported in 28.8% and vital status was known in 98.6%. Of those reporting SGLT2i use at baseline, >80% stayed on SGLT2i for the trial duration (Extended Data Fig. 3). Of those reporting no SGLT2i use at baseline, increasing numbers of participants started SGLT2i during the trial, with a greater proportion starting SGLT2i in the placebo (approximately 10% at 18 months and approximately 20% at 36 months) than in the semaglutide group (approximately 5% at 18 months and approximately 10% at 36 months; Extended Data Fig. 4).

### Outcomes with/without SGLT2i use at baseline

#### Primary outcome

**Fig. 1:**
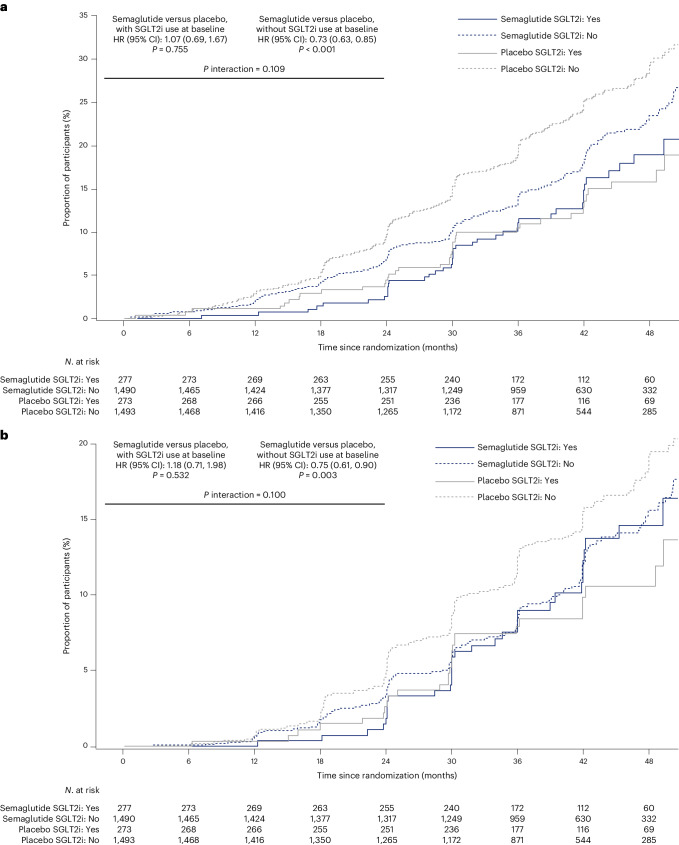
Outcomes for semaglutide 1.0 mg versus placebo in subgroups with/without SGLT2i use at baseline for the primary five-component outcome and for the kidney-specific, four-component outcome. **a**, For the primary five-component outcome, cumulative incidence rates were calculated using the Aalen–Johansen method with non-CV and non-renal death as a competing risk. **b**, For the kidney-specific, four-component outcome (five-component outcome without CV death), cumulative incidence rates were calculated using the Aalen–Johansen method with all-cause death, excluding renal death, as a competing risk. A stratified Cox proportional hazards model was used (stratified by SGLT2i use at baseline (yes/no)), with treatment and subgroup as fixed factors and two-sided *P* values. In **a**, the *P* value for semaglutide versus placebo in participants on SGLT2i at baseline was 0.7546, and for those not on SGLT2i, it was <0.0001; the *P* interaction value was 0.1090.

The incidence of the primary outcome in subgroups with or without SGLT2i use at baseline is displayed in Fig. 1a. During a median follow-up of 3.4 years, in the subgroup reporting use of SGLT2i at baseline, there were 41/277 (14.8%) primary outcomes with semaglutide versus 38/273 (13.9%) in participants with placebo (hazard ratio (HR) 1.07; 95% confidence interval (CI): 0.69, 1.67; *P* = 0.755; Supplementary Table 2). In the subgroup without SGLT2i use at baseline, those numbers were 290/1,490 (19.5%) versus 372/1,493 (24.9%) for semaglutide and placebo groups, respectively (HR 0.73; 95% CI: 0.63, 0.85; *P* < 0.001; *P* interaction 0.109).

The kidney-specific, four-component outcome that excluded CV death (Fig. 1b) occurred in the subgroup with SGLT2i use at baseline in 32/277 (11.6%) participants on semaglutide and 27/273 (9.9%) participants on placebo (HR 1.18; 95% CI: 0.71, 1.98; *P* = 0.532; Supplementary Table 2). In the subgroup without SGLT2i use at baseline, those numbers were 186/1,490 (12.5%) versus 233/1,493 (15.6%) for semaglutide and placebo groups, respectively (HR 0.75; 95% CI: 0.61, 0.90; *P* = 0.003; *P* interaction 0.100).

**Fig. 2:**
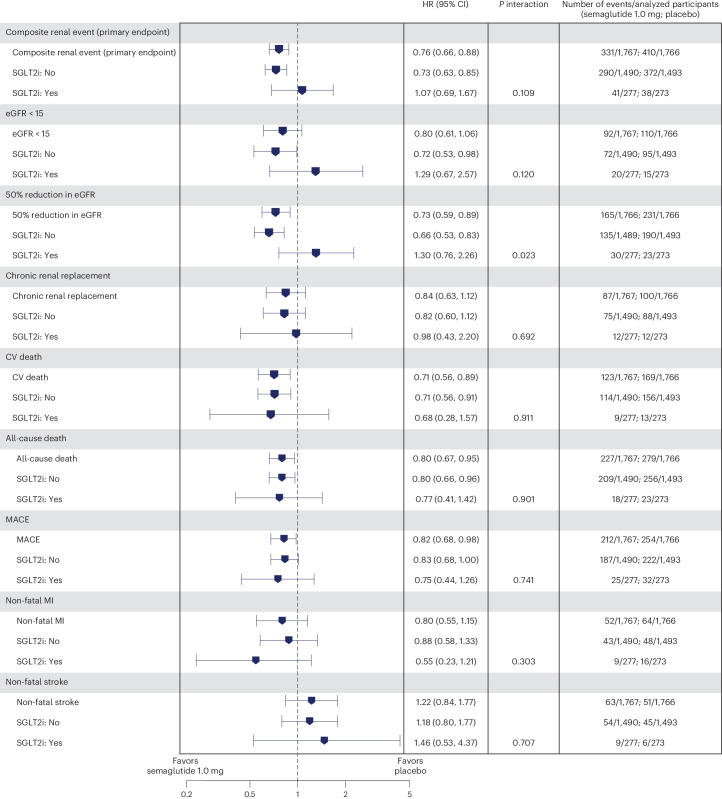
The composite primary outcome and its components, and MACE and all-cause death outcomes. Data from the in-trial period (full analysis set). Data are presented as HR (blue symbol) and corresponding 95% CI (error bars). Time from randomization to relevant endpoint was analyzed using a stratified Cox proportional hazards model with treatment as a categorical fixed factor and two-sided *P* values. Participants without events of interest were censored at the end of their in-trial period. For the subgroup analyses, estimated HR and corresponding CI were calculated in a Cox proportional hazards model with interaction between treatment groups and subgroup as a fixed factor. *P* interaction values for the test of no interaction effect between SGLT2i use and treatment using a score test are shown. There was no renal death in the SGLT2i use subgroup, which is not displayed here. MI, myocardial infarction.

Figure 2 depicts the treatment effects of semaglutide for components of the composite primary outcome in the subgroups.

#### Confirmatory secondary outcomes

##### Decline in eGFR

**Fig. 3:**
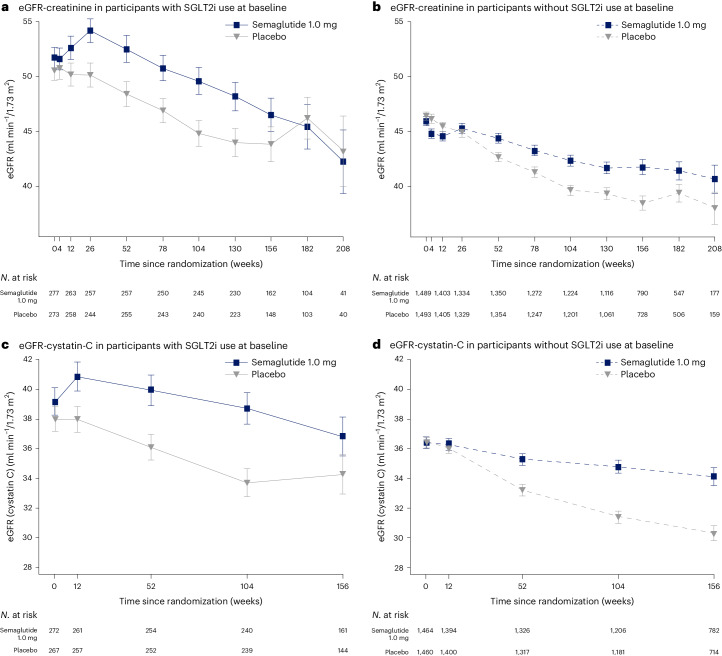
eGFR over time with or without SGLT2i use at baseline, based on serum creatinine and cystatin C. **a**–**d**, Observed data from the in-trial period. eGFR-creatinine in subgroup with SGLT2i use at baseline (**a**), eGFR-creatinine in subgroup without SGLT2i use at baseline (**b**), eGFR-cystatin C in subgroup with SGLT2i use at baseline (**c**) and eGFR-cystatin C in subgroup without SGLT2i use at baseline (**d**). Error bars are ± s.e.m. Numbers shown under the plots represent the number of participants contributing to the means. s.e.m., standard error of the mean.

The changes in eGFR based on serum creatinine over time with or without use of SGLT2i at baseline are displayed in Fig. 3a,b, and based on cystatin C in Fig. 3c,d. The annual rate of decline in eGFR, measured by total eGFR_creat_ slope, in the subgroup with SGLT2i use at baseline was −2.17 (95% CI: −2.70, −1.64) versus −2.92 (95% CI: −3.46, −2.38) ml min^−1^/1.73 m^2^/year with semaglutide versus placebo; that rate in the subgroup without SGLT2i use at baseline was −2.20 (95% CI: −2.43, −1.96) versus −3.44 (95% CI: −3.68, −3.20) ml min^−1^/1.73 m^2^/year with semaglutide versus placebo. The between-group differences of semaglutide versus placebo were 0.75 (95% CI: −0.01, 1.50) and 1.25 (95% CI: 0.91, 1.58) ml min^−1^/1.73 m^2^/year with or without SGLT2i use, respectively (*P* interaction 0.237).

##### MACE; all-cause death

**Extended Data Fig. 5:**
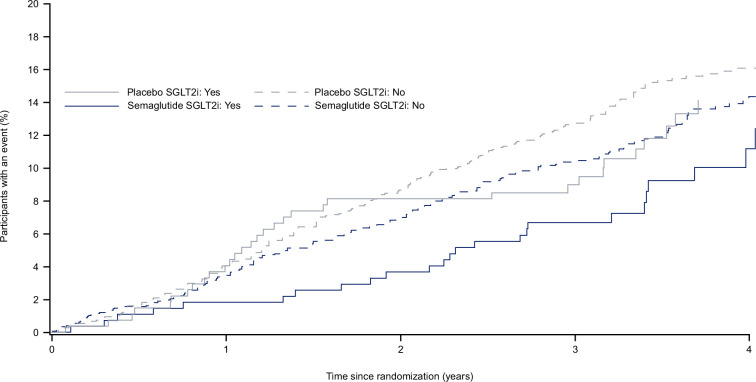
Time to first MACE with semaglutide or placebo according to baseline use of SGLT2i. Cumulative incidence was calculated using the Aalen–Johansen estimator for first MACE accounting for non-CV death as a competing event. CV, cardiovascular; MACE, major adverse CV event (non fatal myocardial infarction, non fatal stroke or CV death); SGLT2i, sodium-glucose co-transporter-2 inhibitors.

The composite major adverse cardiovascular event (MACE) outcome (nonfatal myocardial infarction, nonfatal stroke or CV death) was less frequent with semaglutide versus placebo, and there was no difference between subgroups with or without SGLT2i use at baseline (Fig. 2 and Extended Data Fig. 5; *P* interaction 0.741). Results for the three components of the composite MACE outcome consistently showed interaction *P* values indicating no effect modification by SGLT2i use (*P* = 0.303 to 0.911).

**Extended Data Fig. 6:**
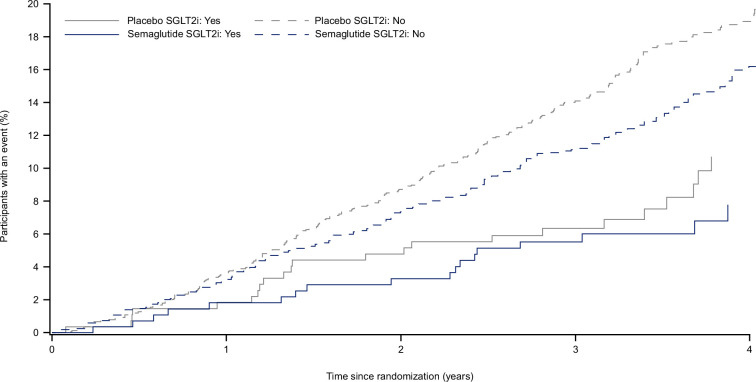
Time to all-cause death with semaglutide or placebo according to baseline use of SGLT2i. Cumulative incidence was calculated using the Aalen–Johansen method for all-cause death. SGLT2i, sodium-glucose co-transporter-2 inhibitors.

All-cause death was less frequent with semaglutide versus placebo, with no difference between those reporting or not reporting SGLT2i use at baseline (Fig. 2 and Extended Data Fig. 6 for time-to-event data; *P* interaction 0.901).

#### Supportive secondary outcomes and safety

**Extended Data Fig. 7:**
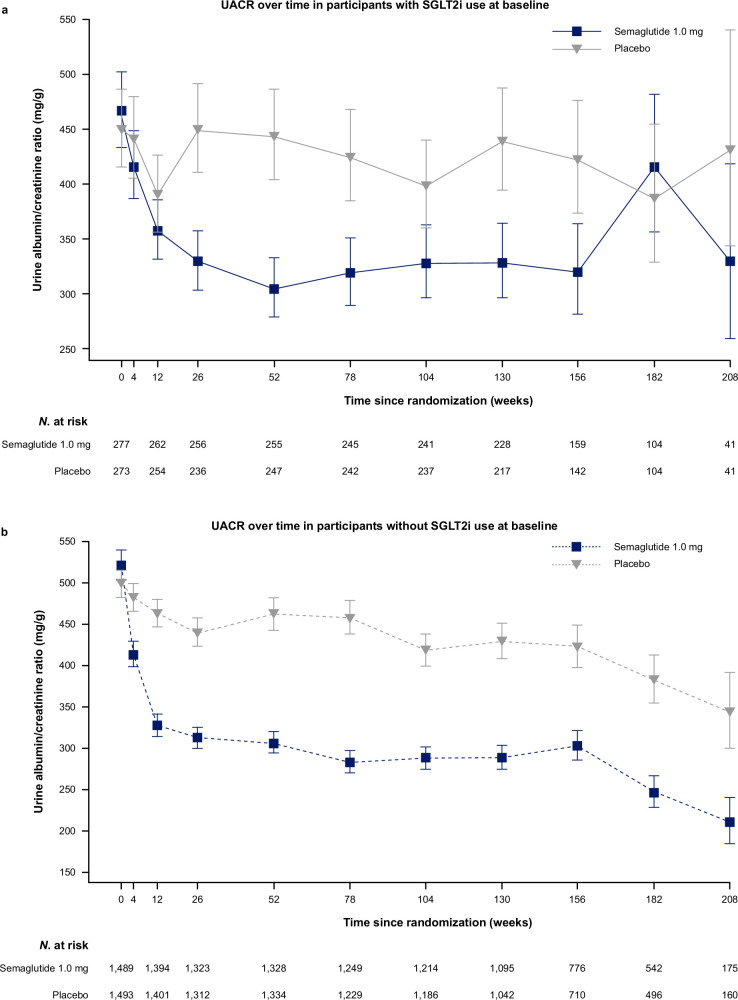
UACR over time in participants with SGLT2i use at baseline (a) or without (b). Observed data from the in-trial period. Error bars are +/– the standard error of the mean on the logarithmic scale back-transformed to natural scale with the exponential. Numbers shown in the lower panel represent the number of participants contributing to the means. SGLT2i, sodium-glucose co-transporter-2 inhibitors; UACR, urine albumin-to-creatinine ratio.

Semaglutide lowered UACR (Extended Data Fig. 7) compared with placebo at week 104; 24% (95% CI: 4%, 39%) and 34% (95% CI: 26%, 40%) in the subgroups with or without SGLT2i use at baseline, respectively (*P* interaction 0.279). HbA_1c_ lowering from baseline to week 104 was greater with semaglutide versus placebo, −8.2 mmol mol^−1^ versus 1.2 mmol mol^−1^ and −9.7 mmol mol^−1^ versus −1.0 mmol mol^−1^ in those with or without SGLT2i use at baseline, respectively (*P* interaction 0.606). Similarly, body weight decreased more with semaglutide compared with placebo, −6.1 kg versus −1.1 kg and −6.3 kg versus −1.5 kg in those with or without SGLT2i use at baseline, respectively (*P* interaction 0.746).

**Extended Data Fig. 8:**
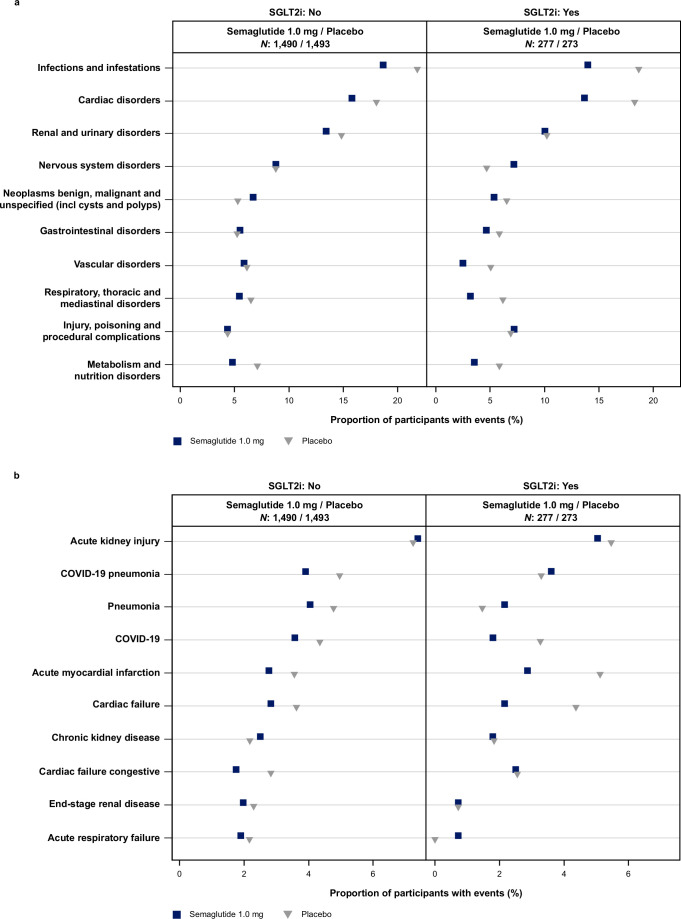
Most frequent serious adverse events by baseline SGLT2i use; by system organ class (a) and by preferred term (b). Percentages are sorted in descending order by the frequency in the semaglutide 1.0 mg group. Most frequent was defined as the 10 most frequent terms by absolute count, where absolute count was >1 in the total population (up to 15 in case of a tie). N, number of participants in the semaglutide and placebo arms. COVID-19, coronavirus disease 2019; SGLT2i, sodium-glucose co-transporter-2 inhibitors. Note that the percentage range (abscissa) varies.

Serious adverse events in the subgroup with SGLT2i use at baseline were reported in 48.4% versus 53.8% of participants receiving semaglutide versus placebo, and 49.9% versus 53.8% in the subgroup without SGLT2i use at baseline. Extended Data Fig. 8 reports details with no differences of safety data between subgroups with/without SGLT2i use at baseline.

#### Initiation of SGLT2i during the trial

Analyzing the primary outcome in participants who reported SGLT2i use at baseline or who initiated an SGLT2i during the study (*n* = 563 in semaglutide and *n* = 658 in placebo groups; Supplementary Table 3), there were 82 (14.6%) and 109 (16.6%) primary outcomes with semaglutide and placebo, respectively, with an HR of 0.88 (95% CI: 0.66, 1.17). The corresponding numbers in those who did not use SGLT2i at baseline or during the study (*n* = 1,208 in semaglutide and *n* = 1,108 in placebo groups) were 249 (20.7%) and 301 (27.2%) primary outcomes, respectively, with an HR of 0.70 (95% CI: 0.59, 0.82; *P* interaction 0.169). When SGLT2i use during the study was treated as a time-dependent covariate in a Cox regression analysis, the HR for the primary outcome was 0.75 (95% CI: 0.65, 0.86). Using a time-dependent Cox regression analysis with SGLT2i use by randomized treatment as a fixed factor, the HRs for the primary outcome were 0.92 (95% CI: 0.64, 1.33) in those using SGLT2i during the study (either from baseline or initiation during study) and 0.72 (95% CI: 0.61, 0.84) in those never using SGLT2i during the study. Supplementary Table 3 shows time-to-event outcomes according to use of SGLT2i at baseline and during study, versus those never on SGLT2i, and Extended Data Fig. 4 depicts the number and time point of new SGLT2i use during the study.

#### Post hoc analyses: eGFR based on cystatin C

For the primary outcome based on changes of eGFR_cystatinC_, without requiring availability of a confirmatory eGFR measurement, HRs for kidney outcomes of semaglutide versus placebo in subgroups with and without use of SGLT2i were overlapping (for example, for the five-component primary outcome: HR 0.74, 95% CI: 0.47, 1.16; and 0.70, 95% CI: 0.60, 0.82, respectively; *P* interaction 0.844), with *P* interaction values of between 0.799 and 0.983 across renal outcomes (Supplementary Table 4).

The annual rate of decline in eGFR, measured by total eGFR_cystatinC_ slope, in the subgroup with SGLT2i use at baseline was −1.04 (95% CI: −1.57, −0.50) versus −1.96 (95% CI: −2.50, −1.42) ml min^−1^/1.73 m^2^/year with semaglutide versus placebo; that rate in the subgroup without SGLT2i use at baseline was −1.30 (95% CI: −1.54, −1.06) versus −2.85 (95% CI: −3.09, −2.61) ml min^−1^/1.73 m^2^/year with semaglutide versus placebo. The between-group differences for semaglutide versus placebo were 0.92 (95% CI: 0.16, 1.68) and 1.55 (95% CI: 1.21, 1.88) ml min^−1^/1.73 m^2^/year less eGFR decline, with or without SGLT2i use, respectively (*P* interaction 0.142).

When the decline in eGFR_creat_ and eGFR_cystatinC_ from baseline was calculated at week 104 after randomization (Table 1), as prespecified, the data were consistent with the slope analysis, that is, loss of eGFR was less with semaglutide than with placebo with no statistical interaction for SGLT2i use. The apparent increase in eGFR at trial end in participants using SGLT2i on placebo, diverging from linear changes, may be due to the low number of observations, survival bias with more deaths in the placebo group, or chance.

**Table 1:** Change in eGFR at week 104 from baseline, based on serum creatinine or cystatin C

	SGLT2i *N* = 550		No SGLT2i *N* = 2,983
**Based on serum creatinine (ml min**^**−1**^**/1.73** **m**^**2**^**)**			
** Semaglutide**	−1.6 (0.77) (−3.1, −0.0) *n* = 268		−4.1 (0.34) (−4.8, −3.4) *n* = 1,381
** Placebo**	−5.3 (0.78) (−6.8, −3.7) *n* = 260		−7.3 (0.35) (−8.0, −6.6) *n* = 1,364
** Between-group difference semaglutide –** **placebo**	3.7 (1.09) (1.6, 5.8)		3.2 (0.49) (2.3, 4.2)
*** P*** **value**	<0.001		<0.001
*** P*** **interaction value**		0.686	
**Based on cystatin C (ml min**^**−1**^**/1.73** **m**^**2**^**)**			
** Semaglutide**	−0.2 (0.67) (−1.6, 1.1) *n* = *263*		−2.4 (0.30) (−2.9, −1.8) *n* = 1,357
** Placebo**	−3.8 (0.67) (−5.1, −2.4) *n* = 254		−5.7 (0.30) (−6.3, −5.1) *n* = 1,333
** Between-group difference semaglutide – placebo**	3.5 (0.95) (1.6, 5.4)		3.4 (0.43) (2.5, 4.2)
*** P*** **value**	<0.001		<0.001
*** P*** **interaction value**		0.901	

Data shown are mean estimates (s.e.) (95% CI) from the in-trial period. *P* interaction values for differences between semaglutide and placebo are shown for the analyses based upon serum creatinine and cystatin C. Responses were analyzed using an ANCOVA with treatment by SGLT2i group as fixed factor and baseline value as covariate. Before analysis, missing data were multiple imputed. The imputation model (linear regression) was done separately for each treatment arm, included baseline value as a covariate, and was fitted to all participants with a measurement regardless of treatment status at week 104. Mean estimates were adjusted according to observed baseline distribution. Two-sided *P* value for test of no treatment difference using a *t*-test within SGLT2i group and an *F*-test for interaction effect between treatment by SGLT2i group. ANCOVA, analysis of covariance; s.e., standard error.

## Discussion

In a prespecified analysis of the FLOW trial, the overall benefits of semaglutide on kidney and CV outcomes in participants with T2D and CKD were not influenced by concomitant use of an SGLT2i. No clear heterogeneity by SGLT2i use was observed for any of the primary or confirmatory secondary outcomes; while power was limited due to the low use of SGLT2i at trial entry, the analyses looking at eGFR slope suggest benefits of semaglutide are observed irrespective of SGLT2i use. The FLOW trial^8^ recently reported that in participants with T2D and CKD, semaglutide 1.0 mg once weekly reduced the risk of major kidney disease events by 24% and blunted eGFR decline by 1.16 ml min^−1^/1.73 m^2^/year^4^. There was also an 18% reduction in MACE and a 20% reduction in all-cause mortality^4^. As individuals with diabetes and CKD are at exceedingly high risk for kidney failure, CV events and death, these results support a clinically meaningful benefit of semaglutide, with or without SGLT2i therapy, for this population.

There are nuances when interpreting the effects of combining SGLT2i with semaglutide in participants with T2D and CKD. The substantial benefits of semaglutide on MACE and mortality did not differ between those treated with or without SGLT2i at baseline. Thus, we suggest that the CV and survival benefits of semaglutide are independent of SGLT2i and possibly additive. For the primary kidney outcome, heterogeneity of semaglutide’s benefits was not detected based on baseline SGLT2i use. This observation must be interpreted with caution for several reasons. The study was not designed to test by specific subgroups, and the power for testing interactions was low because the number of participants using SGLT2i at baseline was small, at just 15.6% of randomized participants. Moreover, the outcomes of kidney disease events occurred later (for example, approximately 5% at 24 months) versus MACE (for example, approximately 5% at 12 months), which also reflects lower power to detect treatment effects on kidney outcomes within the trial time frame. Furthermore, a substantial benefit of semaglutide on eGFR decline with and without SGLT2i use was observed (Table 1 and Fig. 3). Similarly, the decrease in UACR with semaglutide was well preserved in those with baseline SGLT2i use. Therefore, a trial duration of 3.4 median years may be too short to examine kidney outcomes, beyond eGFR and UACR, of combined drug use within the relatively small cohort of 550 participants. When we analyzed the larger subgroup of those using SGLT2i at baseline or who initiated an SGLT2i during the study, kidney-specific benefits (primary outcome) of semaglutide were comparable to those not using SGLT2i, but CIs were overlapping neutrality. A recent observational analysis of a register from the United Kingdom investigated chronic users of GLP-1 RAs that started SGLT2i medication as well as chronic users of SGLT2i starting GLP-1 RAs^9^. The authors concluded that the combination of both drug classes was associated with a lower risk of MACE and serious kidney disease events compared with either drug class alone. That conclusion is supported by other reviews and meta-analyses^10,11^.

In the three kidney outcomes trials examining SGLT2i, the use of GLP-1 RAs was rare, below 5%^12–14^. Only one of these three trials, to our knowledge, published data on the GLP-1 RA subgroup (*n* = 122 of 4,304) and found no interaction of GLP-1 RA use with SGLT2i benefits on main outcomes^12^. A recent meta-analysis of all major SGLT2i outcome trials focused on 3,065/72,970 participants using GLP-1 RAs (4%) simultaneously with SGLT2i/placebo^15^. The authors concluded that CV and kidney benefits of SGLT2i were independent of background use of GLP-1 RAs. There are numerous CV outcome trials in T2D that examined either GLP-1 RAs or SGLT2i. However, a history of CKD was rare in those trials and combined use (non-randomized and non-stratified) of both drug classes was reported in 0–5.3%^16^. Lam et al. analyzed the AMPLITUDE-O trial, which examined a GLP-1 RA versus placebo and stratified for SGLT2i use (618 participants, 15.2%)^16^. Overall, in this general T2D cohort, the benefits of GLP-1 RAs on CV and kidney outcomes were not impacted by SGLT2i use. However, there was a strong trend for SGLT2i to enhance albuminuria and heart failure benefits, but blunting benefits on myocardial infarction and stroke of the GLP-1 RA in AMPLITUDE-O^16^. Similar data stem from a CV outcomes trial that examined an SGLT2i versus placebo, DECLARE-TIMI 58 (Dapagliflozin Effect on Cardiovascular Events–Thrombolysis in Myocardial Infarction 58)^17^. Several smaller short-term trials reported additive effects of GLP-1 RAs and SGLT2i on surrogate outcomes such as body weight, blood pressure and HbA_1c_^18–21^.

SGLT2i represent a cornerstone in the management of T2D with CKD^22^. Outcomes trials confirmed that SGLT2i effectively prevented kidney failure as well as heart failure events, with more modest effects on atherosclerotic CV outcomes^3^. Semaglutide, on the other hand, impacts particularly atherosclerotic CV and kidney outcomes as well as mortality, as now shown with the FLOW trial^1^. Therefore, the combination of semaglutide with SGLT2i may be complementary for kidney and heart protection in T2D with CKD.

Our analysis has limitations, mainly the limited power in the groups with baseline SGLT2i use. Thus, we may have not detected small but clinically relevant interactions but the data do not refute independent actions of these drug classes. Also, the post-baseline initiations of SGLT2i therapy were not controlled and more participants initiated SGLT2i in the placebo group. We did not find safety concerns of combining semaglutide with SGLT2i in participants with T2D and CKD. To learn more about combining semaglutide with SGLT2i, an outcomes trial comparing both drugs with their combination would be of clear importance for patient care. Potential strengths of FLOW include its consistent enrollment and retention during the coronavirus disease 2019 pandemic, the high adherence to randomized treatment (89%) and the lower-than-expected number of dropouts.

In conclusion, semaglutide reduced risks of kidney, CV and all-cause mortality outcomes without heterogeneity of those benefits by SGLT2i use in participants with T2D and CKD. Given the substantial benefits of both semaglutide and SGLT2i, and the acceptable safety profile of their combination, this option may be considered when treating patients with T2D and CKD.

## Methods

The design and main results of this randomized, double-blind, placebo-controlled, international, multicenter kidney outcomes trial have been detailed previously^4,8^; the trial protocol is available alongside the FLOW primary publication^4^. The trial was overseen by an academic-led steering committee in partnership with the sponsor Novo Nordisk, who managed trial operations and funded editorial assistance for this report. The first draft of this manuscript was written by the first author, with all authors contributing to subsequent revisions who had access to the full dataset.

All participants provided written informed consent, and the protocol was approved by both national and institutional ethical and regulatory authorities.

In brief, participants with T2D and CKD were randomly assigned double-blind in a 1:1 ratio to receive semaglutide 1 mg per week subcutaneously or matching placebo using a central interactive web response system between June 2019 and May 2021. SGLT2i use was permitted, and randomization was stratified by SGLT2i use at baseline (yes/no). Included were participants with an eGFR of 25−75 ml min^−1^/1.73 m^2^ (calculated from serum creatinine using the Chronic Kidney Disease Epidemiology^23^ formula) and UACR of >300 to <5,000 mg g^−1^ if the eGFR was ≥50 ml min^−1^/1.73 m^2^, or a UACR of >100 to <5,000 mg g^−1^ if the eGFR was 25 to < 50 ml min^−1^/1.73 m^2^, while receiving a stable maximal labeled or tolerated dose of RASi. Individuals intolerable to RASi were eligible for inclusion. Up to 20% of participants were allowed to have an eGFR of ≥60 ml min^−1^/1.73 m^2^.

The full list of inclusion/exclusion criteria are:

Inclusion criteria:

- Informed consent obtained before any trial-related activities. Trial-related activities are any procedures that are carried out as part of the trial, including activities to determine suitability for the trial, except for protocol-described prescreening activities, which require a separate informed consent
- Male or female, age above or equal to 18 years at the time of signing informed consent
- Diagnosed with T2D mellitus
- HbA_1c_ ≤10% (≤86 mmol mol^−1^)^a^
- Renal impairment defined either by:
  - Serum creatinine-based eGFR of ≥50 and ≤75 ml min^−1^/1.73 m^2^ (CKD-EPI)^a^, ^b^ and UACR of >300 and <5,000 mg g^−1^ ^a^
    *or*
  - Serum creatinine-based eGFR of ≥25 and <50 ml min^−1^/1.73 m^2^ (CKD-EPI)^a^ and UACR of >100 and <5,000 mg g^−1^ ^a^
- Treatment with maximum labeled or tolerated dose of a renin–angiotensin–aldosterone system (RAAS) blocking agent including an angiotensin-converting enzyme inhibitor or an angiotensin II receptor blocker, unless such treatment is contraindicated or not tolerated. Treatment dose must have been stable for at least 4 weeks before the date of the laboratory assessments used for determination of inclusion criterion 5 and kept stable until screening

^a^ Laboratory results for inclusion were based on:

- Measurements no more than 90 days old at screening, documented in medical records
  or
- Measurements from the optional prescreening visit, documented in medical records
  or
- Central laboratory measurement obtained at the screening visit

The participant must have been in their usual health condition at the time of sample collection used for inclusion as evaluated by the investigator and treated with an RAAS blocking agent.

^b^The number of participants with an inclusion eGFR of ≥60 ml min^−1^/1.73 m^2^ was capped at 20% of randomized participants.

Exclusion criteria:

- Known or suspected hypersensitivity to trial product(s) or related products
- Previous participation in this trial; participation was defined as randomization
- Female who is pregnant, is breastfeeding, intends to become pregnant or is of childbearing potential and not using a highly effective contraceptive method
- Participation in any clinical trial of an approved or non-approved investigational medicinal product within 30 days before screening^a^
- Any disorder, which in the investigator’s opinion might have jeopardized the participant’s safety or compliance with the protocol
- Congenital or hereditary kidney diseases including polycystic kidney disease, autoimmune kidney diseases including glomerulonephritis or congenital urinary tract malformations
- Use of any GLP-1 RAs within 30 days before screening
- Personal or first-degree relative(s) history of multiple endocrine neoplasia type 2 or medullary thyroid carcinoma
- Myocardial infarction, stroke, hospitalization for unstable angina pectoris or transient ischemic attack within 60 days before the day of screening
- Presently classified as being in the New York Heart Association class IV of heart failure
- Planned coronary, carotid or peripheral artery revascularization
- Current (or within 90 days) chronic or intermittent hemodialysis or peritoneal dialysis
- Uncontrolled and potentially unstable diabetic retinopathy or maculopathy. Verified by a fundus examination performed within the past 90 days before screening or in the period between screening and randomization. Pharmacological pupil dilation is a requirement unless using a digital fundus photography camera specified for non dilated examination
- Presence or history of malignant neoplasm within 5 years before the day of screening. Basal and squamous cell skin cancer and any carcinoma in situ are allowed
- A prior solid organ transplant or awaiting solid organ transplant
- Combination use of an angiotensin-converting enzyme inhibitor and an angiotensin II receptor blocker

The primary outcome was a five-component composite of onset of a ≥50% reduction in eGFR from the baseline value sustained for at least 28 days of kidney failure (commencement of chronic dialysis, kidney transplantation or a reduction in eGFR to <15 ml min^−1^/1.73 m^2^ sustained for at least 28 days) or death due to kidney or CV causes. The first value was used for an eGFR of <15 ml min^−1^/1.73 m^2^ or 50% reduction. For eGFR at baseline, the means of screening eGFR and eGFR at randomization were used. If only one of these values was available, that number was used. Serum creatinine was measured in a central laboratory and eGFR calculated using the Chronic Kidney Disease Epidemiology^23^ formula.

Three key confirmatory secondary outcomes were assessed using hierarchical testing if superiority was confirmed for the primary outcome. The first was rate of loss of kidney function, defined as total eGFR slope (annual rate of change in eGFR); the second was time to first MACE (composite of nonfatal myocardial infarction, nonfatal stroke or CV death), and the third was death due to any cause. Other outcomes were prespecified as exploratory (see the FLOW protocol in ref. ^8^). We also tested a four-component composite outcome, identical to the primary outcome but excluding death due to CV causes.

We also measured serum cystatin C centrally at randomization, at week 12 and week 52, and then on a yearly basis after randomization until year 3, and calculated eGFR accordingly to calculate rate of loss of kidney function and total eGFR slope. From these measurements, we post hoc derived the five-point and four-point kidney endpoints without persistence confirmed for eGFR values.

A prespecified single interim analysis was triggered in October 2023 after approximately two-thirds (570) of the planned total primary outcomes had accrued. The Independent Data Monitoring Committee reviewed the data and recommended early completion of the trial for efficacy. This recommendation was accepted and the trial was completed.

### Statistical analysis

This trial was event driven and designed to provide 90% power to detect a 20% relative risk reduction for semaglutide versus placebo for the primary outcome^8^. Assuming an event rate for the primary outcome of 7.5% per year in the placebo group, a minimum 3,508 participants were to be enrolled in the trial, requiring a minimum of 854 primary endpoint events. An interim analysis for efficacy was planned after two-thirds of the total planned number of primary outcome events had occurred.

Efficacy analyses were based on the intention-to-treat principle and were to include all unique participants who underwent randomization irrespective of adherence to semaglutide or placebo or changes to background medications. Data from participants who withdrew from the trial, died from causes not included in the primary endpoint, or were lost to follow-up were censored at the time of withdrawal, death or last contact with the investigator. Time-to-event endpoints were analyzed using a stratified Cox proportional hazards model with randomized treatment group (semaglutide or placebo) as a fixed factor. Subgroup analyses were performed adding the interaction term between SGLT2i use at baseline and treatment group. Likewise, time-to-event outcomes were plotted by randomized treatment group and SGLT2i use at baseline using the Aalen–Johansen estimator and presented as cumulative incidences considering non-CV death/non-renal death or all-cause death as a competing event dependent of the outcome. Furthermore, two time-dependent Cox regression models were performed; one with SGLT2i use as the time-dependent variable (yes/no) and randomized treatment as a fixed factor, and another with SGLT2i use interacting with randomized treatment.

The total and chronic (from week 12) eGFR slopes were analyzed using a linear random regression model with randomized treatment group, SGLT2i use at baseline (yes/no), time and treatment by time interaction as fixed effects, participant as a random intercept and time as a random slope. Missing data for scheduled eGFR values were not imputed. Subgroup analyses were also performed adding the interaction term: treatment by time by SGLT2i use at baseline, to assess if there were different treatment slopes by SGLT2i use.

Continuous supportive secondary endpoints: changes in eGFR and other continuous endpoints (changes from baseline to week 104) were assessed by analysis of covariance with treatment by SGLT2i use at baseline (yes/no) adjusted for the relevant continuous endpoint at baseline. Multiple imputations were used for missing values under a missing-at-random assumption. Results were combined using Rubin’s rule. Interaction *P* values were derived from an *F*-test of equality between the treatment differences across the SGLT2i use. Log transformation was applied before analysis for parameters specified in the statistical analysis plan, and treatment differences were expressed as a treatment mean ratio.

No adjustment for multiplicity or alpha-protection was performed. Two-sided *P* values below 0.05 were considered significant. All statistical analyses were performed with SAS software, version 9.4 (SAS Institute). Novo Nordisk maintained the clinical database and performed the statistical analyses.

### Reporting summary

Further information on research design is available in the Nature Portfolio Reporting Summary linked to this article.

## Online content

Any methods, additional references, Nature Portfolio reporting summaries, source data, extended data, supplementary information, acknowledgements, peer review information; details of author contributions and competing interests; and statements of data and code availability are available at 10.1038/s41591-024-03133-0.

## Supplementary information

Supplementary Information Supplementary Tables 1–4

Reporting Summary

## Data Availability

Data will be shared with bona fide researchers who submit a research proposal approved by the independent review board. Individual participant data will be shared in datasets in a de-identified and anonymized format. Data will be made available after research completion and approval of the product and product use in the European Union and the United States. Information about data access request proposals can be found at https://www.novonordisk-trials.com/.
