# Supplementary material for: Effects of semaglutide with and without concomitant SGLT2 inhibitor use in participants with type 2 diabetes and chronic kidney disease in the FLOW trial
Source: Nat Med. 2024 Jun 24;30(10):2849–56. doi: 10.1038/s41591-024-03133-0 (PMC11485243; doi:10.1038/s41591-024-03133-0)
Supplement: Supplementary file 1 — Supplementary Tables 1–4 [file 41591_2024_3133_MOESM1_ESM.pdf]

nature medicine

Supplementary information https://doi.org/10.1038/s41591-024-03133-0

Effects of semaglutide with and without concomitant SGLT2 inhibitor use in participants with type 2 diabetes and chronic kidney disease in the FLOW trial

In the format provided by the authors and unedited

Content

Supplementary Table 1 | Clinical characteristics and demographics in participants using SGLT2i at baseline.......... 2

Supplementary Table 2 | Outcomes in participants according to SGLT2i use (with/without) at baseline 6

Supplementary Table 3 | Outcomes in participants using SGLT2i at baseline or during the trial or not using SGLT2i at any time.......... 10

Supplementary Table 4 | Cox regression of renal outcomes with eGFR calculated with cystatin C – with or without SGLT-2i use at baseline, a post-hoc analysis.......... 13

Supplementary Table 1 | Clinical characteristics and demographics in participants using SGLT2i at baseline

	**Semaglutide 1.0 mg**			**Placebo**		
**Data are reported as *n* (%), unless otherwise indicated**	Overall (*N* = 1,767)	SGTL2i use (*N* = 277)	SGTL2i no use (*N* = 1,490)	Overall (*N* = 1,766)	SGTL2i use (*N* = 273)	SGTL2i no use (*N* = 1,493)
**Age (mean [SD], years)**	66.6 (9.0)	64.7 (8.7)	66.9 (9.0)	66.7 (9.0)	64.9 (9.7)	67.0 (8.8)
**Sex**						
**Female**	519 (29.4)	61 (22.0)	458 (30.7)	550 (31.1)	63 (23.1)	487 (32.6)
**Region**						
**Asia**	478 (27.1)	108 (39.0)	370 (24.8)	434 (24.6)	96 (35.2)	338 (22.6)
**Europe**	472 (26.7)	72 (26.0)	400 (26.8)	491 (27.8)	60 (22.0)	431 (28.9)
**North America**	423 (23.9)	64 (23.1)	359 (24.1)	442 (25.0)	69 (25.3)	373 (25.0)
**Other**	394 (22.3)	33 (11.9)	361 (24.2)	399 (22.6)	48 (17.6)	351 (23.5)
**Race**						
**White**	1,155 (65.4)	156 (56.3)	999 (67.0)	1,168 (66.1)	164 (60.1)	1,004 (67.2)
**Asian**	439 (24.8)	103 (37.2)	336 (22.6)	407 (23.0)	95 (34.8)	312 (20.9)
**Black or African American**	78 (4.4)	5 (1.8)	73 (4.9)	82 (4.6)	7 (2.6)	75 (5.0)

**Other[a]**	95 (5.4)	13 (4.7)	82 (5.5)	109 (6.2)	7 (2.6)	102 (6.8)
**Ethnicity**						
**Hispanic or Latino**	273 (15.4)	21 (7.6)	252 (16.9)	283 (16.0)	36 (13.2)	247 (16.5)
**Not Hispanic or Latino**	1,421 (80.4)	248 (89.5)	1,173 (78.7)	1,411 (79.9)	232 (85.0)	1,179 (79.0)
**Not reported**	73 (4.1)	8 (2.9)	65 (4.4)	72 (4.1)	5 (1.8)	67 (4.5)
**HbA_{1c} (mean [SD], %)**	7.8 (1.3)	7.8 (1.2)	7.8 (1.3)	7.8 (1.3)	7.8 (1.2)	7.8 (1.3)
**Body mass index (mean [SD], kg/m^2)**	31.9 (6.1)	32.0 (6.0)	31.9 (6.1)	32.0 (6.5)	31.4 (6.4)	32.1 (6.6)
**Body weight (mean [SD], kg)**	89.5 (19.8)	91.3 (20.1)	89.1 (19.7)	89.8 (21.2)	89.0 (21.1)	90.0 (21.3)
**Systolic blood pressure (mean [SD], mmHg)**	138.9 (16.1)	135.4 (16.4)	139.5 (16.0)	138.4 (15.4)	134.5 (14.8)	139.1 (15.4)
**Diastolic blood pressure (mean [SD], mmHg)**	76.8 (10.0)	75.9 (9.6)	76.9 (10.1)	76.1 (10.0)	74.6 (9.6)	76.4 (10.1)
**Diabetes duration (years)**						
**<15**	774 (43.8)	128 (46.2)	646 (43.4)	753 (42.6)	113 (41.4)	640 (42.9)
**≥15**	992 (56.1)	149 (53.8)	843 (56.6)	1,013 (57.4)	160 (58.6)	853 (57.1)
**Prior MI or stroke**						

**Yes**	405 (22.9)	59 (21.3)	346 (23.2)	403 (22.8)	56 (20.5)	347 (23.2)
**Chronic heart failure**						
**Yes**	342 (19.4)	42 (15.2)	300 (20.1)	336 (19.0)	40 (14.7)	296 (19.8)
**Tobacco use**						
**Current smoker**	223 (12.6)	43 (15.5)	180 (12.1)	206 (11.7)	35 (12.8)	171 (11.5)
**Never smoked**	883 (50.0)	120 (43.3)	763 (51.2)	864 (48.9)	116 (42.5)	748 (50.1)
**Previous smoker**	661 (37.4)	114 (41.2)	547 (36.7)	696 (39.4)	122 (44.7)	574 (38.4)
**eGFR (mean [SD], ml/min/1.73m^2)**	46.9 (15.6)	51.7 (15.5)	46.0 (15.5)	47.1 (14.7)	50.6 (15.2)	46.5 (14.5)
**Renal function, eGFR (ml/min/1.73m^2)**						
**≥60**	366 (20.7)	82 (29.6)	284 (19.1)	353 (20.0)	69 (25.3)	284 (19.0)
**≥45 to <60**	515 (29.1)	95 (34.3)	420 (28.2)	540 (30.6)	103 (37.7)	437 (29.3)
**≥30 to <45**	667 (37.7)	79 (28.5)	588 (39.5)	691 (39.1)	79 (28.9)	612 (41.0)
**<30**	218 (12.3)	21 (7.6)	197 (13.2)	182 (10.3)	22 (8.1)	160 (10.7)
**UACR (median, mg/g)**	582.3	556.1	589.0	557.8	491.1	563.4
**Albuminuria (category)**						

**A1 (normoalbuminuria <30 mg/g)**	52 (2.9)	8 (2.9)	44 (3.0)	57 (3.2)	8 (2.9)	49 (3.3)
**A2 (microalbuminuria ≥30 to <300 mg/g)**	509 (28.8)	94 (33.9)	415 (27.9)	495 (28.0)	85 (31.1)	410 (27.5)
**A3 (macroalbuminuria ≥300 mg/g)**	1,205 (68.2)	175 (63.2)	1,030 (69.1)	1,214 (68.7)	180 (65.9)	1,034 (69.3)

For eGFR, the baseline assessment is defined as the mean of the two assessments from the randomization visit and the screening visit. Albuminuria categories are based on UACR, and the baseline assessment is defined as the mean of the two assessments from the randomization visit. If only one of the assessments for either UACR or eGFR is available, this is used as the baseline assessment. The renal function categories are based on the eGFR as per CKD-EPI. % values show the percentage of participants in the full analysis set. For all parameters except UACR and eGFR, baseline is defined as the eligible assessment associated with the randomization visit, if taken before or at the date of first dose. If missing or taken after the date of first dose, the assessment from the screening visit is used. Smoking is defined as smoking at least one cigarette or equivalent daily. [a]Includes participants whose race was reported as “American Indian or Alaska Native”, “Native Hawaiian or Other Pacific Islander”, or “Not reported”. CKD-EPI, Chronic Kidney Disease Epidemiology Collaboration; eGFR, estimated glomerular filtration rate; HbA_{1c}, glycated hemoglobin; IQR, interquartile range; MI, myocardial infarction; SD standard deviation; SGLT2i, sodium-glucose co-transporter-2 inhibitors; UACR, urine albumin-to-creatinine ratio.

Supplementary Table 2 | Outcomes in participants according to SGLT2i use (with/without) at baseline

	Semaglutide						Semaglutide vs placebo				
	Total (*N* = 3,533) N (%) [IR]		1.0 mg (*N* = 1,767) N (%) [IR]		Placebo (*N* = 1,766) N (%) [IR]		HR (95% CI)	P	HR (95% CI)	P	*P*-inter.
Baseline SGLT2i use:	Yes	No	Yes	No	Yes	No	Yes		No		
Subgroup n	550	2,983	277	1,490	273	1,493					
5-component outcome	79 (14.4) [4.4]	662 (22.2) [7.1]	41 (14.8) [4.6]	290 (19.5) [6.1]	38 (13.9) [4.3]	372 (24.9) [8.1]	1.07 (0.69, 1.67)	0.755	0.73 (0.63, 0.85)	<0.001	0.109
4-component kidney outcome	59 (10.7) [3.3]	419 (14.0) [4.5]	32 (11.6) [3.6]	186 (12.5) [3.9]	27 (9.9) [3.0]	233 (15.6) [5.1]	1.18 (0.71, 1.98)	0.532	0.75 (0.61, 0.90)	0.003	0.100

Onset of persistent ≥50% reduction in eGFR	53 (9.6) [3.0]	325 (10.9) [3.5]	30 (10.8) [3.3]	135 (9.1) [2.8]	23 (8.4) [2.6]	190 (12.7) [4.1]	1.30 (0.76, 2.26)	0.347	0.66 (0.53, 0.83)	<0.001	0.023
Onset of persistent eGFR <15 ml/min/1.73 m^2	35 (6.4) [1.9]	167 (5.6) [1.8]	20 (7.2) [2.2]	72 (4.8) [1.5]	15 (5.5) [1.7]	95 (6.4) [2.0]	1.29 (0.67, 2.57)	0.452	0.72 (0.53, 0.98)	0.039	0.120
RRT	24 (4.4) [1.3]	163 (5.5) [1.7]	12 (4.3) [1.3]	75 (5.0) [1.5]	12 (4.4) [1.3]	88 (5.9) [1.9]	0.98 (0.43, 2.20)	0.954	0.82 (0.60, 1.12)	0.212	0.692
Onset of persistent eGFR <15 ml/min/1.73 m^2 or RRT	42 (7.6) [2.3]	265 (8.9) [2.8]	23 (8.3) [2.5]	119 (8.0) [2.5]	19 (7.0) [2.1]	146 (9.8) [3.1]	1.18 (0.64, 2.19)	0.598	0.78 (0.61, 0.99)	0.045	0.216
Renal death	0 (0.0) [0.0]	10 (0.3) [0.1]	0 (0.0) [0.0]	5 (0.3) [0.1]	0 (0.0) [0.0]	5 (0.3) [0.1]	NA	NA	0.97 (0.28, 3.35)	0.963	–

MACE	57	409	25	187	32	222	0.75	0.287	0.83	0.054	0.741
	(10.4)	(13.7)	(9.0)	(12.6)	(11.7)	(14.9)	(0.44, 1.26)		(0.68, 1.00)		
	[3.2]	[4.3]	[2.7]	[3.9]	[3.6]	[4.7]					
Death from	22	270	9	114	13	156	0.68	0.368	0.71	0.006	0.911
CV causes	(4.0)	(9.1)	(3.2)	(7.7)	(4.8)	(10.4)	(0.28, 1.57)		(0.56, 0.91)		
	[1.2]	[2.8]	[1.0]	[2.3]	[1.4]	[3.2]					
Non-fatal MI	25	91	9	43	16	48	0.55	0.147	0.88	0.547	0.303
	(4.5)	(3.1)	(3.2)	(2.9)	(5.9)	(3.2)	(0.23, 1.21)		(0.58, 1.33)		
	[1.4]	[0.9]	[1.0]	[0.9]	[1.8]	[1.0]					
Non-fatal stroke	15	99	9	54	6	45	1.46	0.469	1.18	0.401	0.707
	(2.7)	(3.3)	(3.2)	(3.6)	(2.2)	(3.0)	(0.53, 4.37)		(0.80, 1.77)		
	[0.8]	[1.0]	[1.0]	[1.1]	[0.7]	[0.9]					
All-cause death	41	465	18	209	23	256	0.77	0.397	0.80	0.016	0.901
	(7.5)	(15.6)	(6.5)	(14.0)	(8.4)	(17.1)	(0.41, 1.42)		(0.66, 0.96)		
	[2.2]	[4.8]	[1.9]	[4.2]	[2.5]	[5.3]					

The analysis was based on a Cox proportional hazards model containing an interaction term for treatment by subgroup, with treatment and subgroup as fixed factors and two-sided *P*-values; the HRs in the treatment part of the table compare semaglutide 1.0 mg to placebo within each subgroup. *P*-interaction values for the test of no interaction effect between SGLT2i use and treatment using a score test are shown. The 5-component outcome was a composite of onset of a sustained ≥50% reduction in eGFR from the baseline value, of kidney failure (commencement of chronic dialysis, kidney transplantation, or a reduction in eGFR to <15 ml/min/1.73m^2 sustained for at least 28 days), or death due to kidney- or CV causes. The 4-component kidney outcome excluded death due to CV causes. MACE was a composite of non-fatal MI, non-fatal stroke, or CV death. CI, confidence interval; CKD, chronic kidney disease; CV, cardiovascular; eGFR, estimated glomerular filtration rate; HR, hazard ratio; IR, incidence rate (events/100 patient years of observation); MACE, major adverse cardiovascular event; MI, myocardial infarction; *P*, *P*-value; *P*-inter, *P*-interaction value; RRT, renal-replacement therapy; SGLT2i, sodium-glucose cotransporter-2 inhibitors.

Supplementary Table 3 | Outcomes in participants using SGLT2i at baseline or during the trial or not using SGLT2i at any time

	**Total (*N* = 3,533)**	**SGLT2i use (Yes vs no)**		**Semaglutide 1.0 mg (*N* = 1,767)**	**Placebo (*N* = 1,766)**	**Treatment semaglutide vs placebo**		
	N (%) [IR]	HR (95% CI)	P	N (%) [IR]	N (%) [IR]	HR (95% CI)	**P**	***P*-inter.**
**Participants using SGLT2i at baseline or during the trial**								
**Subgroup n**	1,221			563	658			
**5-component outcome**	191 (15.6) [4.7]			82 (14.6) [4.4]	109 (16.6) [5.0]	0.88 (0.66, 1.17)	0.381	0.169
**4-component kidney outcome**	141 (11.5) [3.5]			64 (11.4) [3.4]	77 (11.7) [3.5]	0.97 (0.69, 1.35)	0.853	0.110

**MACE**	128			49	79	0.71	0.062	0.466
	(10.5)			(8.7)	(12.0)	(0.49, 1.01)		
	[3.2]			[2.6]	[3.6]			
**All-cause death**	91			34	57	0.69	0.087	0.638
	(7.5)			(6.0)	(8.7)	(0.45, 1.05)		
	[2.2]			[1.7]	[2.5]			
**Participants not using SGLT2i at any time**								
**Subgroup n**	2,312			1,204	1,108			
**5-component**	550	1.69	<0.001	249	301	0.70	<0.001	0.169
**outcome**	(23.8)	(1.38, 2.08)		(20.7)	(27.2)	(0.59, 0.82)		
	[7.8]			[6.6]	[9.2]			
**4-component**	337	1.42	0.005	154	183	0.70	0.001	0.109
**kidney outcome**	(14.6)	(1.12, 1.82)		(12.8)	(16.5)	(0.57, 0.87)		
	[4.8]			[4.1]	[5.6]			
**MACE**	338	1.53	0.001	163	175	0.83	0.088	0.466
	(14.6)	(1.19, 1.99)		(13.5)	(15.8)	(0.67, 1.03)		
	[4.7]			[4.3]	[5.1]			

**All-cause death**	415<br>(17.9)<br>[5.6]	2.73<br>(2.06, 3.71)	<0.001	193<br>(16.0)<br>[4.9]	222<br>(20.0)<br>[6.3]	0.77<br>(0.64, 0.93)	0.0083	0.638

The analysis was based on a Cox proportional hazards model containing an interaction term for treatment by subgroup, with treatment and subgroup as fixed factors and two-sided *P*-values; the HRs in the treatment part of the table compare semaglutide 1.0 mg to placebo within each subgroup. *P*-interaction values for the test of no interaction effect between SGLT2i use and treatment using a score test are shown. The 5-component outcome was a composite of onset of a sustained ≥50% reduction in eGFR from the baseline value, of kidney failure (commencement of chronic dialysis, kidney transplantation, or a reduction in eGFR to <15 ml/min/1.73m^2 sustained for at least 28 days), or death due to kidney- or CV causes. The 4-component kidney outcome excluded death due to CV causes. MACE was a composite of non-fatal MI, non-fatal stroke, or CV death. CI, confidence interval; CKD, chronic kidney disease; CV, cardiovascular; eGFR, estimated glomerular filtration rate; HR, hazard ratio; IR, incidence rate (events/100 patient years of observation); MACE, major adverse cardiovascular event; MI, myocardial infarction; *P*, *P*-value; *P*-inter, *P*-interaction value; SGLT2i, sodium-glucose cotransporter-2 inhibitors.

Supplementary Table 4 | Cox regression of renal outcomes with eGFR calculated with cystatin C – with or without SGLT-2i use at baseline, a post-hoc analysis

	**Total (*N* = 3,533)** N (%) [IR]	**SGLT2i use (yes vs no)** HR (95% CI)	P	**Semaglutide 1.0 mg (*N* = 1,767)** N (%) [IR]	**Placebo (*N* = 1,766)** N (%) [IR]	**Treatment semaglutide vs placebo** HR (95% CI)	**P**	***P*- inter.**
**Participants using SGLT2i at baseline**								
**Subgroup n**	550			277	273			
**5-component CKD with eGFR cystatin**	76 (13.8) [4.3]			33 (11.9) [3.6]	43 (15.8) [4.9]	0.74 (0.47, 1.16)	0.189	0.844
**4-component CKD with eGFR cystatin**	58 (10.5) [3.3]			24 (8.7) [2.6]	34 (12.5) [3.9]	0.68 (0.40, 1.14)	0.149	0.919
**Onset of ≥50% reduction in eGFR cystatin**	19 (3.5) [1.1]			7 (2.5) [0.8]	12 (4.4) [1.4]	0.56 (0.21, 1.40)	0.228	0.799
**Onset of eGFR cystatin <15 ml/min/1.73 m^2**	40 (7.3) [2.2]			16 (5.8) [1.8]	24 (8.8) [2.7]	0.64 (0.34, 1.20)	0.172	0.983

Participants not using SGLT2i at baseline

**Subgroup n**	2983			1490	1493			
**5-component CKD with eGFR cystatin**	652 (21.9) [7.1]	1.67 (1.33, 2.14)	<0.001	277 (18.6) [5.9]	375 (25.1) [8.3]	0.70 (0.60, 0.82)	<0.001	0.844
**4-component CKD with eGFR cystatin**	427 (14.3) [4.6]	1.43 (1.10, 1.91)	0.010	181 (12.1) [3.9]	246 (16.5) [5.5]	0.70 (0.58, 0.85)	<0.001	0.919
**Onset of ≥50% reduction in eGFR cystatin**	197 (6.6) [2.1]	2.03 (1.30, 3.36)	0.003	68 (4.6) [1.4]	129 (8.6) [2.9]	0.50 (0.37, 0.66)	<0.001	0.799
**Onset of eGFR cystatin <15 ml/min/1.73 m^2**	232 (7.8) [2.5]	1.11 (0.80, 1.57)	0.549	93 (6.2) [2.0]	139 (9.3) [3.0]	0.65 (0.50, 0.84)	0.001	0.983

The post-hoc analysis of eGFR was based on measurement of cystatin C in serum which was done at randomization, and at week 12 and week 52, and then every 12 months after randomization (see Fig. 3). When $eGFR_{cystatinC}$ was beyond thresholds (>50% decrease or below 15 ml/min/1.73m^2), no confirmatory measurement was done. Supplementary Table 2 displays eGFR based on serum creatinine which was measured at least every 6 months and confirmatory measurements were done when above thresholds were met. The analysis was based on a stratified Cox proportional hazards model containing an interaction term for treatment by subgroup, with treatment and subgroup as fixed factors and two-sided *P*-values, stratified by SGLT2i use at baseline; the HRs in the treatment part of the table compare semaglutide 1.0 mg to placebo within each subgroup. *P*-interaction values for the test of no interaction effect between SGLT2i use and

treatment using a score test are shown. The 5-component outcome was a composite of onset of a ≥50% reduction in eGFR (calculated from serum cystatin C) from the baseline value, of kidney failure (commencement of chronic dialysis sustained for at least 28 days, kidney transplantation, or a reduction in eGFR to <15 ml/min/1.73m^2 (calculated from serum cystatin C), or death due to kidney or CV causes. The 4-component kidney outcome excluded death due to CV causes. CI, confidence interval; CKD, chronic kidney disease; CV, cardiovascular; eGFR, estimated glomerular filtration rate; HR, hazard ratio; IR, incidence rate (events/100 patient years of observation); *P*, *P*-value; *P*-inter, *P*-interaction value; SGLT2i, sodium-glucose cotransporter-2 inhibitors.
