# Supplementary material for: Effects of semaglutide with and without concomitant SGLT2 inhibitor use in participants with type 2 diabetes and chronic kidney disease in the FLOW trial
Source: Nat Med. 2024 Jun 24;30(10):2849–56. doi: 10.1038/s41591-024-03133-0 (PMC11485243; doi:10.1038/s41591-024-03133-0)
Supplement: Supplementary file 2 — Reporting Summary [file 41591_2024_3133_MOESM2_ESM.pdf]

Corresponding author(s): Professor Johannes F.E. Mann

Last updated by author(s): Jun 11, 2024

Reporting Summary

Nature Portfolio wishes to improve the reproducibility of the work that we publish. This form provides structure for consistency and transparency in reporting. For further information on Nature Portfolio policies, see our Editorial Policies and the Editorial Policy Checklist.

Statistics

For all statistical analyses, confirm that the following items are present in the figure legend, table legend, main text, or Methods section.

n/a	Confirmed	
☐	☒	The exact sample size (*n*) for each experimental group/condition, given as a discrete number and unit of measurement
☒	☐	A statement on whether measurements were taken from distinct samples or whether the same sample was measured repeatedly
☐	☒	The statistical test(s) used AND whether they are one- or two-sided<br>*Only common tests should be described solely by name; describe more complex techniques in the Methods section.*
☐	☒	A description of all covariates tested
☒	☐	A description of any assumptions or corrections, such as tests of normality and adjustment for multiple comparisons
☐	☒	A full description of the statistical parameters including central tendency (e.g. means) or other basic estimates (e.g. regression coefficient) AND variation (e.g. standard deviation) or associated estimates of uncertainty (e.g. confidence intervals)
☐	☒	For null hypothesis testing, the test statistic (e.g. F, t, r) with confidence intervals, effect sizes, degrees of freedom and P value noted<br>*Give P values as exact values whenever suitable.*
☒	☐	For Bayesian analysis, information on the choice of priors and Markov chain Monte Carlo settings
☒	☐	For hierarchical and complex designs, identification of the appropriate level for tests and full reporting of outcomes
☐	☒	Estimates of effect sizes (e.g. Cohen's *d*, Pearson's *r*), indicating how they were calculated

Our web collection on statistics for biologists contains articles on many of the points above.

Software and code

Policy information about availability of computer code

Data collection	No software was used for data collection.
Data analysis	All statistical analyses were performed with SAS software, version 9.4 (SAS Institute, Cary, NC, USA).

For manuscripts utilizing custom algorithms or software that are central to the research but not yet described in published literature, software must be made available to editors and reviewers. We strongly encourage code deposition in a community repository (e.g. GitHub). See the Nature Portfolio guidelines for submitting code & software for further information.

Data

Policy information about availability of data

All manuscripts must include a data availability statement. This statement should provide the following information, where applicable:

- Accession codes, unique identifiers, or web links for publicly available datasets
- A description of any restrictions on data availability
- For clinical datasets or third party data, please ensure that the statement adheres to our policy

Data will be shared with bona fide researchers who submit a research proposal approved by the independent review board. Individual patient data will be shared in datasets in a de-identified and anonymized format. Data will be made available after research completion and approval of the product and product use in the European Union and the United States. Information about data access request proposals can be found at https://www.novonordisk-trials.com/.

Research involving human participants, their data, or biological material

Policy information about studies with human participants or human data. See also policy information about sex, gender (identity/presentation), and sexual orientation and race, ethnicity and racism.

Reporting on sex and gender	Biologic sex was reported for trial participants; information on gender was not collected
Reporting on race, ethnicity, or other socially relevant groupings	Race and ethnicity were reported for trial participants
Population characteristics	Participants aged 18 years and above, with type 2 diabetes and chronic kidney disease, as reported in Supplementary Table 1
Recruitment	Participants were recruited if they met the eligibility criteria.
Ethics oversight	All patients provided written informed consent, and the protocol was approved by both national and institutional ethical and regulatory authorities.

Note that full information on the approval of the study protocol must also be provided in the manuscript.

Field-specific reporting

Please select the one below that is the best fit for your research. If you are not sure, read the appropriate sections before making your selection.

☒ Life sciences ☐ Behavioural & social sciences ☐ Ecological, evolutionary & environmental sciences

For a reference copy of the document with all sections, see nature.com/documents/nr-reporting-summary-flat.pdf

Life sciences study design

All studies must disclose on these points even when the disclosure is negative.

Sample size	This trial was event driven and designed to provide 90% power to detect a 20% relative risk reduction for semaglutide versus placebo for the primary outcome2. Assuming an event rate for the primary outcome of 7.5% per year in the placebo group, a minimum 3,508 participants were to be enrolled in the trial, requiring a minimum of 854 primary endpoint events.
Data exclusions	No data were excluded
Replication	N/A
Randomization	Eligible participants were randomly assigned 1:1 to receive semaglutide or matching placebo using a central interactive web response system
Blinding	This was a double-blind study, neither participants nor investigators were aware of the treatment being administered.

Reporting for specific materials, systems and methods

We require information from authors about some types of materials, experimental systems and methods used in many studies. Here, indicate whether each material, system or method listed is relevant to your study. If you are not sure if a list item applies to your research, read the appropriate section before selecting a response.

Materials & experimental systems

n/a	Involved in the study	
☒	☐	Antibodies
☒	☐	Eukaryotic cell lines
☒	☐	Palaeontology and archaeology
☒	☐	Animals and other organisms
☐	☒	Clinical data
☒	☐	Dual use research of concern
☒	☐	Plants

Methods

n/a	Involved in the study	
☒	☐	ChIP-seq
☒	☐	Flow cytometry
☒	☐	MRI-based neuroimaging

Clinical data

Policy information about clinical studies

All manuscripts should comply with the ICMJE guidelines for publication of clinical research and a completed CONSORT checklist must be included with all submissions.

Clinical trial registration	NCT03819153
Study protocol	Published in previous papers
Data collection	Investigators were responsible for data collection; recruitment occurred between June 2019 and May 2021, and data were collected from 387 sites across 28 countries, with the last participant visit being January 9, 2024.
Outcomes	In this secondary analysis, clinical outcomes of semaglutide versus placebo were assessed in participants with type 2 diabetes and chronic kidney disease, who were or were not taking SGLT-2 inhibitors at baseline. The primary outcome was a composite of major kidney disease events, defined as onset of kidney failure (commencement of chronic dialysis, kidney transplantation, or a reduction in eGFR to <15 ml/min/1.73m2 sustained for at least 28 days), a sustained (at least 28 days) ≥50% reduction in eGFR from baseline, or death due to kidney- or cardiovascular causes. Three key secondary outcomes were defined and assessed: total eGFR slope (annual rate of change in eGFR from randomisation to end-of-trial); time to first major cardiovascular event (composite of non-fatal myocardial infarction, non-fatal stroke, or cardiovascular death), and death due to any cause.

Plants

Seed stocks	*Report on the source of all seed stocks or other plant material used. If applicable, state the seed stock centre and catalogue number. If plant specimens were collected from the field, describe the collection location, date and sampling procedures.*
Novel plant genotypes	*Describe the methods by which all novel plant genotypes were produced. This includes those generated by transgenic approaches, gene editing, chemical/radiation-based mutagenesis and hybridization. For transgenic lines, describe the transformation method, the number of independent lines analyzed and the generation upon which experiments were performed. For gene-edited lines, describe the editor used, the endogenous sequence targeted for editing, the targeting guide RNA sequence (if applicable) and how the editor was applied.*
Authentication	*Describe any authentication procedures for each seed stock used or novel genotype generated. Describe any experiments used to assess the effect of a mutation and, where applicable, how potential secondary effects (e.g. second site T-DNA insertions, mosiacism, off-target gene editing) were examined.*
